# Perceptions of Caregiving: Racial Examinations of Resiliency and Stress

**DOI:** 10.1093/geroni/igaa057.1133

**Published:** 2020-12-16

**Authors:** Athena Koumoutzis, Kelly Cichy

**Affiliations:** 1 Miami University, Oxford, Ohio, United States; 2 Kent State University, Kent, Ohio, United States

## Abstract

Adult children are at risk of emotional strain when parental caregiving needs emerge. Pearlin’s Stress Process Model (1990) and caregiver studies suggest minority caregivers report lower subjective caregiving burden, however, few studies simultaneously consider both the stresses and rewards of caregiving. Using data from Wave II of the Family Exchange Study (N = 243), we examine racial differences in midlife adults’ perceptions (i.e., stress and rewards) of assisting their parents with activities of daily living (ADLs) and the associations between perceptions of ADL assistance and emotional well-being among adults who help their parents with ADLs. Compared to non-minority caregivers (M = 4.18, SD = 0.91), minority caregivers (M = 4.45, SD = 0.84) found it more rewarding to help their mother (t(314) = -2.54, p < .05), whereas non-minority caregivers (M = 2.25, SD = .1.27) found it more stressful to help their father than did minority caregivers (M = 1.64, SD = 0.99), t(162) = 3.01, p < .01). After controlling for demographics and ADL needs, linear regression analyses revealed that the stress of helping parents predicted depression (F(6, 189) = 5.30, p < .001) and race moderated the association (p < .01); the association was only significant for minority caregivers (p < .05). Implications will be discussed.

